# Fabrication and Characterization of Visible to Near-Infrared Photodetector Based on Multilayer Graphene/Mg_2_Si/Si Heterojunction

**DOI:** 10.3390/nano12183230

**Published:** 2022-09-17

**Authors:** Hong Yu, Rui Deng, Zhangjie Mo, Shentong Ji, Quan Xie

**Affiliations:** 1 College of Physics and Electronic Science, Guizhou Education University, Guiyang 550018, China; 2 The College of Big Data and Information Engineering, Guizhou University, Guiyang 550025, China

**Keywords:** MLG, MLG/Mg_2_Si/Si heterojunction, PDs, detection properties

## Abstract

In this investigation, p–Mg_2_Si/n–Si heterojunction photodetector (PD) is fabricated by magnetron sputtering and low vacuum annealing in the absence of argon or nitrogen atmosphere. Multilayer Graphene (MLG)/Mg_2_Si/Si heterojunction PD is first fabricated by transferring MLG to Mg_2_Si/Si heterojunction substrate using the suspended self-help transfer MLG method. After characterizing the phase composition, morphology and detection properties of Mg_2_Si/Si and MLG/Mg_2_Si/Si heterojunction PDs, the successful fabrication of the Mg_2_Si/Si and MLG/Mg_2_Si/Si heterojunction PDs are confirmed and some detection capabilities are realized. Compared with the Mg_2_Si/Si heterojunction PD, the light absorption and the ability to effectively separate and transfer photogenerated carriers of MLG/Mg_2_Si/Si heterojunction PD are improved. The responsivity, external quantum efficiency (EQE), noise equivalent power (NEP), detectivity (D*), on/off ratio and other detection properties are enhanced. The peak responsivity and EQE of the MLG/Mg_2_Si/Si heterojunction PD are 23.7 mA/W and 2.75%, respectively, which are better than the previous 1–10 mA/W and 2.3%. The results illustrate that the fabrication technology of introducing MLG to regulate the detection properties of the Mg_2_Si/Si heterojunction PD is feasible. In addition, this study reveals the potential of MLG to enhance the detection properties of optoelectronic devices, broadens the application prospect of the Mg_2_Si/Si-based heterojunction PDs and provides a direction for the regulation of optoelectronic devices.

## 1. Introduction

Mg_2_Si is an indirect band-gap environmentally friendly semiconductor material extensively studied in thermoelectric materials [1,2], battery materials [3,4], structural materials [5] and composite materials [6,7]. C, Mg and Si are non-toxic, non-polluting and rich in the crust, showing environmentally friendly and sustainable development characteristics. Mg_2_Si has a band gap width of 0.6–0.8 eV [8] and a high absorption coefficient of more than 10^5^ cm^−1^ near 500 nm [9]. Its detection wavelength range is 400–1500 nm, which can extend the optical response cutoff wavelength of the existing Si PD to more than 1100 nm [10]. It exhibits great potential to become a substitute for other toxic, harmful and high-cost heterojunction PDs for visible and near-infrared (NIR) light detection.

In 2007, Atanassov et al. [11] obtained Mg_2_Si using ion beam synthesis technology and attained a band gap of 1.01 eV for Mg_2_Si through infrared and Raman spectra. In 2013, Udono et al. [12] prepared a p–Mg_2_Si/n–Mg_2_Si diode by thermal diffusion. The spectral response range of the p–n junction diode was 1.2–2 μm. At 300 K, the Eg value of the p–n junction was about 0.6 eV. In 2019, El-Amir et al. [13] obtained an n–Mg_2_Si/p–Si diode by the magnetron sputtering co-sputtering method following annealing under argon atmosphere. The diode showed clear rectification characteristics, and the Mg_2_Si film showed strong optical absorption near the wavelength of 1 μm. In 2020, Shevlyqgin et al. [14] synthesized the Mg_2_Si/Si heterojunction by molecular beam epitaxy (MBE) and studied its application in photovoltaics. It was observed that the responsivity value of Mg_2_Si/Si heterojunction from visible to the near-infrared band is 1–10 mA/W, and the spectral wavelength range is 400–1400 nm. This is important for developing Mg_2_Si-based optoelectronic devices. These facts make the Mg_2_Si-based heterojunction a Si-based optoelectronic material. Although Mg_2_Si is an attractive Si-based optoelectronic material, only a few reports focus on Mg_2_Si-based heterojunction PDs.

Graphene (Gr), as a two-dimensional (2D) material, has excellent properties, such as high mobility, low resistivity, tunable Fermi level [15,16] and is used to form a suitable heterojunction with other semiconductor materials. The fact that the band gap will be opened by increasing the number of Gr layers changes the properties of Gr from metallic behavior to narrow bandgap semiconductors, thereby extending the photodetection to the infrared range [17]. By increasing the number of MLG, the absorbance will be boosted up [18]. The resistivity of Gr is about 10^−6^ Ω·cm. Therefore, as the MLG layers increase, the resistivity of the device is decreased and the photocurrent by the device is increased [18]. The high carrier-mobility of MLG also reduces the recombination rate of photogenerated free-charges, which may result in improved optical properties of MLG/InGaAs/InAlAs/InAs [19]. With the addition of MLG, a high built-in electric field is formed at the MLG/Mg_2_Si interface, which is beneficial to the effective separation and transfer of photogenerated carriers, thus improving the detection properties of the device [20].

Using the excellent properties of MLG to regulate the performance characteristics of MLG/semiconductor heterojunction devices, MLG is first introduced to construct the MLG/Mg_2_Si/Si heterojunction PD. Through the characterization of its phase composition, structural morphology, Raman spectrum and other detection properties, the MLG/Mg_2_Si/Si heterojunction PD has been successfully obtained, and sufficient optical absorption and the ability of effective separation and transfer of photogenerated carriers have been improved. Compared with the Mg_2_Si/Si heterojunction PD, the responsivity, EQE, NEP, D*, on/off ratio and other detection properties of MLG/Mg_2_Si/Si heterojunction PD are significantly enhanced.

## 2. Experimental Details and Methods

N–type Si (111) substrate was used in the experiment, with a resistivity of 0.01–0.05 Ω·cm, purity of 99.99%, size of 15 × 15 mm and thickness of 0.5 mm. The cleaning process of Si substrate was as follows: ultrasonic cleaning in acetone solution for 20 min; removing organic matter on the surface; and then rinsing with deionized water. After ultrasonic cleaning in absolute ethanol for 20 min, acetone on the surface was removed and then rinsed with deionized water. Ultrasonic cleaning was carried out in deionized water for 20 min and finally dried in the oven. Both the Si and Mg target had a purity of 99.99%, a diameter in 60 mm and a thickness of 5 mm, and were cleaned with absolute ethanol. A total of 6–8 layers of MLG with the size of 1 × 1 cm were purchased from Hefei Microcrystalline Materials Technology Co., Ltd (Heifei, Chnia). and Jiangsu Xianfeng Nanomaterials Technology Co., Ltd (Nanjing, Chnia).

In the previous study, using a suitable magnetron sputtering and annealing process without argon or nitrogen atmosphere, high-quality single-phase Mg_2_Si films were obtained [21,22,23,24]. Among them, the Mg sputtering process utilized the following conditions: the vacuum at the back of the sputtering chamber was 6.0 × 10^−5^ Pa, the sputtering pressure was 3.0 Pa, the sputtering power was 100 W, the sputtering time was 25 min and the argon flow rate was 30 sccm. Meanwhile, the conditions for the Si sputtering process were: the vacuum at the back and bottom of the sputtering chamber was 6.0 × 10^−5^ Pa, the sputtering pressure was 2.0 Pa, the sputtering power was 110 W, the sputtering time was 25 min, the argon flow rate was 30 sccm and the sputtering bias voltage is 50 V. Then, for the annealing process, the conditions were: low vacuum (10^−1^–10^−2^ Pa), in the absence of argon or nitrogen atmosphere, and the annealing temperature was 400 °C for 4 h.

First, a p–type Si layer was deposited on the n–type Si substrate, and the Mg film was deposited on p–type Si. Mg/p–Si/n–Si samples were annealed under a low vacuum without argon or nitrogen atmosphere. In the annealing process, the impurities in the p-type Si entered the undoped Mg layer so that the annealed Mg_2_Si layer showed p–type characteristics; thus, the p–Mg_2_Si/n–Si heterojunction can be obtained. The Hall effect of the Mg_2_Si/Si heterojunction was examined. The Hall coefficient of the Mg_2_Si layer was positive, and its value was about 4 × 10^−4^ m^3^·c^−1^, opposite to the polarity of the n–type Si substrate, thus forming a p–Mg_2_Si/n–Si heterojunction.

The MLG layer was transferred to the p–Mg_2_Si/n–Si heterojunction substrate by the suspended self-help transfer MLG method, and the MLG/Mg_2_Si/Si heterojunction was prepared. An electrode mask plate was placed on the surface of MLG, the Al film was deposited on the mask plate for 10 min and the Al film was deposited on the back of the Si substrate for 10 min. The electrode preparation was completed by annealing at 400 °C for 20 min. The electrode and probe were connected by conductive adhesive during the test. The main steps of the suspended self-help transfer MLG method were as follows: (1) Releasing MLG in deionized water; (2) Transferring MLG to Mg_2_Si/Si substrate; (3) Drying at room temperature for about 20 min; (4) Baking the sample at 70 °C for 30 min on the electric heating station; (5) Drying the samples at room temperature, immersing in acetone for 10 min, again immersing in another acetone solution for 30 min, and then finally dried.

The HL5500PC Hall effect tester (Nanometrics, Ottawa, ON, Canada) was used to characterize the Hall coefficient of the samples. The film’s crystal structure was tested by an Empyrean X-ray diffractometer (XRD) from PANalytical, the Netherlands. The Su–8010 field emission scanning electron microscopy (FESEM, Hitachi, Tokyo, Japan) was used to observe the surface morphology of the experimental samples. The LabRAM HR Evolution Raman spectrometer (Horiba, Jobin Yvon, Paris, France) was used to characterize the samples for the qualitative molecular structure analysis. The DSR100 PD detection property test system (Beijing Zhuoli Hanguang Co., Ltd., Beijing, China) measured the spectral response, responsivity and EQE of the PD. The model of the digital source meter was Keithley 2614B.

## 3. Results and Discussion

**Figure 1:**
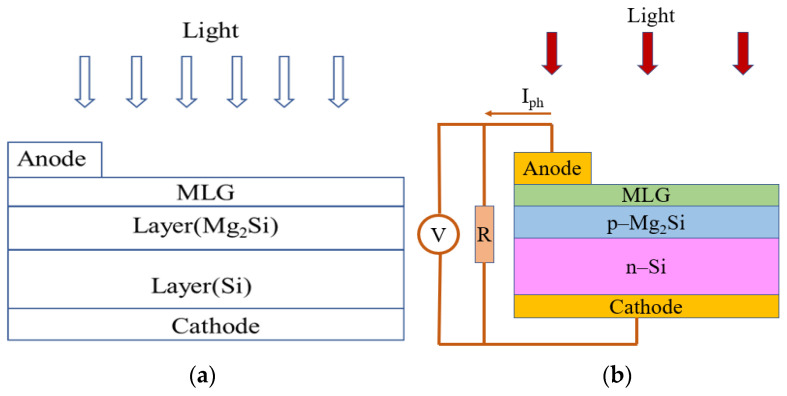
(**a**) The schematic of the experimental fabrication of the MLG/Mg_2_Si/Si PD; (**b**) the measurement circuit of detection properties of the MLG/Mg_2_Si/Si PD.

When the incident light is irradiated on the MLG/Mg_2_Si/Si heterojunction surface, the holes in the Mg_2_Si layer absorb photon energy and then transition to the conduction band, thereby generating electron-hole pairs. Under the built-in electric field, the electron and hole pairs are effectively separated and transferred. The holes follow the direction of the built-in electric field through the transport layer of MLG and the anode and finally reach the external circuit. The electrons then pass through the Mg_2_Si and Si layers to the cathode along the opposite direction of the built-in electric field and finally enter the external circuit, thus realizing the photovoltaic effect. The schematic and measurement circuit of the experimental fabrication of the MLG/Mg_2_Si/Si heterojunction PD is shown in Figure 1.

### 3.1. XRD Characterization 

**Figure 2:**
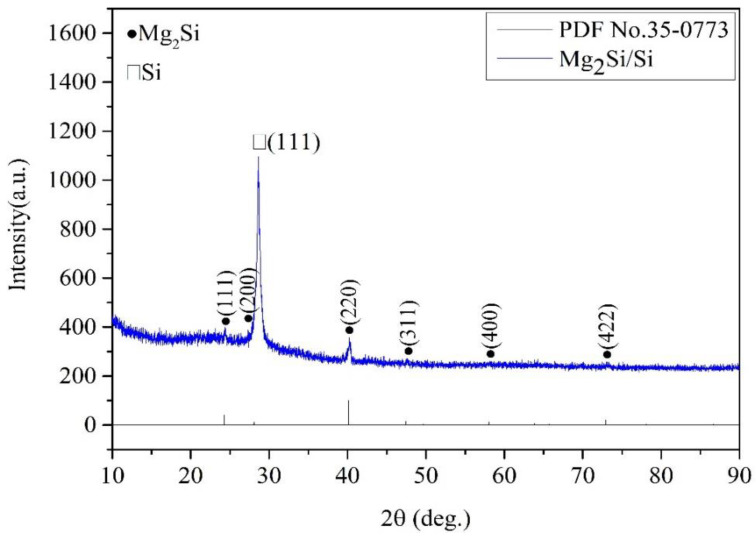
The Mg_2_Si crystal diffraction standard card and XRD spectra of p–Mg_2_Si/n–Si heterojunction.

Figure 2 shows the XRD of the p–Mg_2_Si/n–Si heterojunction after annealing at 400 °C for 4 h under a low vacuum (10^−1^–10^−2^ Pa) without argon or nitrogen atmosphere. It can be seen that the positions of (111), (200), (220), (311), (400) and (422) of Mg_2_Si are consistent with the Mg_2_Si standard card, along with the Si (111) diffraction peak. In addition, there is no residual Mg diffraction peak, indicating the initial formation of the Mg_2_Si/Si heterojunction structure.

### 3.2. Raman Characterization

**Figure 3:**
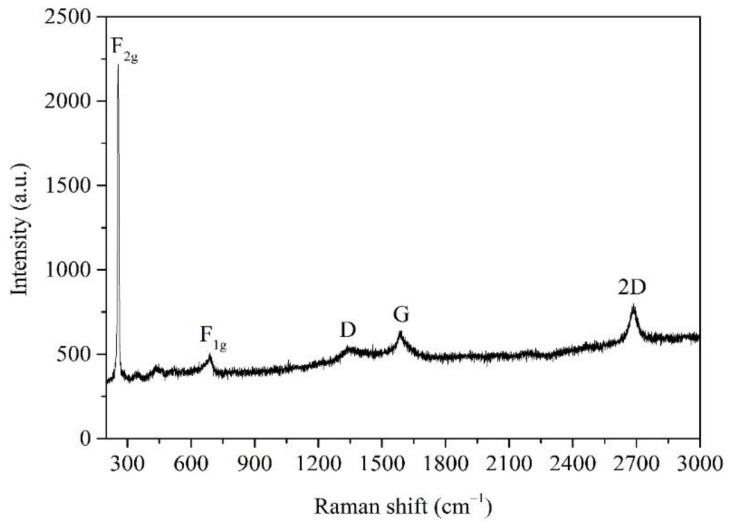
The Raman spectrum of MLG/Mg_2_Si/Si heterojunction.

Figure 3 shows the Raman spectrum of the MLG/Mg_2_Si/Si heterojunction. The D, G and 2D characteristic peaks of MLG are observed around 1350, 1580 and 2680 cm^−1^, respectively, consistent with the Raman characteristic peaks of MLG [25,26,27]. F_2g_ and F_1g_ are the Raman characteristic peaks of Mg_2_Si, located near 256 and 690 cm^−1^, respectively, consistent with the Raman characteristic peaks of Mg_2_Si [21,22,28]. The Raman characteristic peaks of Mg_2_Si and MLG are consistent with their standard Raman characteristic peaks, indicating the formation of the MLG/Mg_2_Si/Si heterojunction structure.

### 3.3. FESEM Characterization

**Figure 4:**
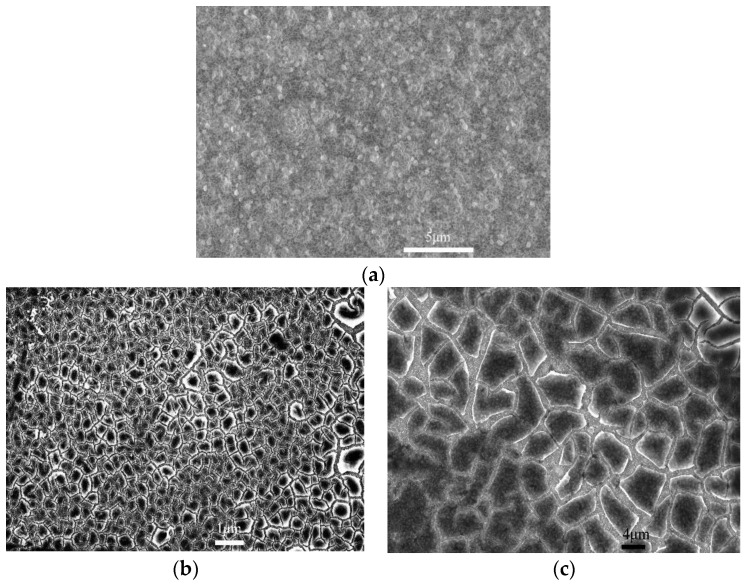
The FESEM images of Mg_2_Si/Si and MLG/Mg_2_Si/Si heterojunctions. (**a**) the FESEM images of Mg_2_Si/Si; (**b**) the FESEM images of MLG/Mg_2_Si/Si at a magnification of 1000 times; (**c**) the FESEM images of MLG/Mg_2_Si/Si at a magnification of 2000 times.

Figure 4 shows the FESEM images of Mg_2_Si/Si and MLG/Mg_2_Si/Si heterojunctions. Figure 4a is obtained by observing the surface of Mg_2_Si with a magnification of 10,000 times. It can be seen that the Mg_2_Si layer has a good crystallization effect, smooth surface, clear grain outline and uniform distribution. Figure 4b is the surface FESEM image of MLG/Mg_2_Si/Si heterojunction at a magnification of 1000 times, and Figure 4c is the surface FESEM image of MLG/Mg_2_Si/Si heterojunction at a magnification of 2000 times. It can be noted that the individual carbon atoms are arranged in a hexagonal shape [29], and the light and dark contrasts in the image correspond to the carbon atoms and the gaps, respectively. Figure 4c exhibits the crystal grains of the Mg_2_Si layer below the MLG layer.

### 3.4. Responsivity

**Figure 5:**
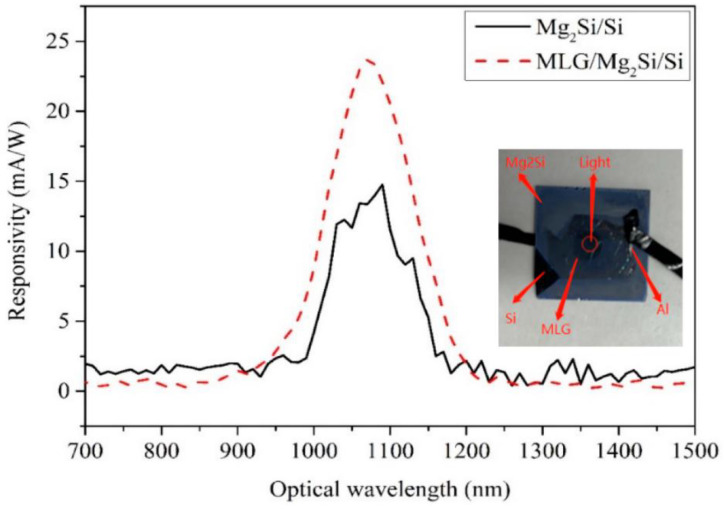
The responsivity curves of Mg_2_Si/Si and MLG/Mg_2_Si/Si PDs.

The experimentally measured responsivity curves of Mg_2_Si/Si and MLG/Mg_2_Si/Si heterojunction PDs are shown in Figure 5. The responsivity is expressed in Equation (1) [30,31], where *I_photo_* is the output photocurrent, *P_incident_* is the incident light power and the responsivity unit is A/W.

(1) $$\;\;Responsivity=\frac{I_{photo}}{P_{incident}}$$

The responsivity is measured at about 0.2 mW/cm^2^ illumination. The bias voltage is 10 V. It can be seen from Figure 5 that the peak responsivity of Mg_2_Si/Si heterojunction PD is 14.76 mA/W, and the peak responsivity of MLG/Mg_2_Si/Si heterojunction PD is 23.7 mA/W. The peak wavelengths are around 1090 nm, and the response wavelength range is 700–1500 nm. Based on this equation, the photocurrents corresponding to the peak responsivity of 14.76 mA/W and 23.7 mA/W are 2.95 μA and 4.74 μA, respectively. Compared with the peak responsivity of the Mg_2_Si/Si heterojunction PD, the peak responsivity of the MLG/Mg_2_Si/Si heterojunction PD is enhanced by about 60%. Shevlyqgin et al. [14] observed that the Mg_2_Si/Si heterojunction has a responsivity of 1–10 mA/W from the visible light to the NIR band, and the results of this study are already better than this level of responsivity.

**Figure 6:**
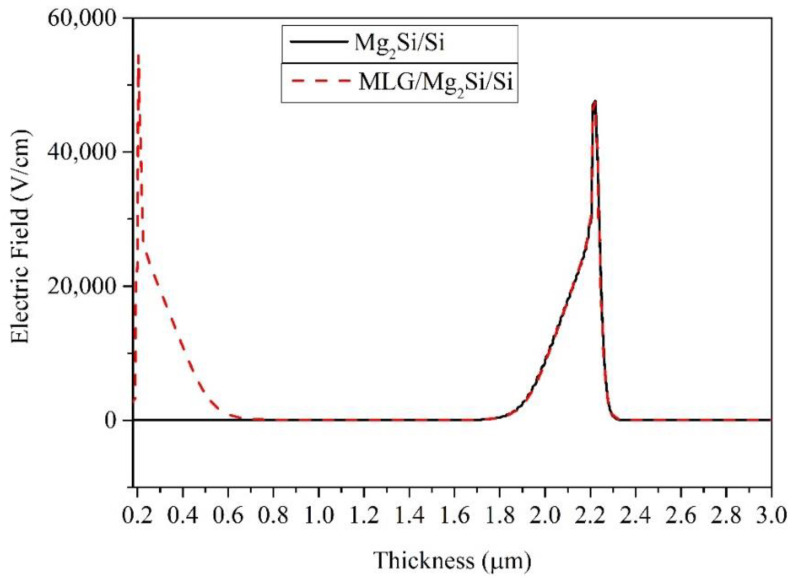
The electric field of Mg_2_Si/Si and MLG/Mg_2_Si/Si PDs.

With the addition of MLG, the light absorption of PD increases, thereby increasing the photocurrent [32]. This is mainly due to the decrease in the MLG/Semiconductor contact resistance [20]. The resistivity of graphene is about 10^−6^ Ω·cm. Therefore, after adding one or more layers of Gr, the resistivity of the device decreases and the photocurrent of the device increases [20]. A high built-in electric field is formed at the MLG/Mg_2_Si interface, which improves the effective separation and transfer of photogenerated carriers under illumination. It increases the responsivity, as shown in Figure 6 [20].

### 3.5. External Quantum Efficiency

**Figure 7:**
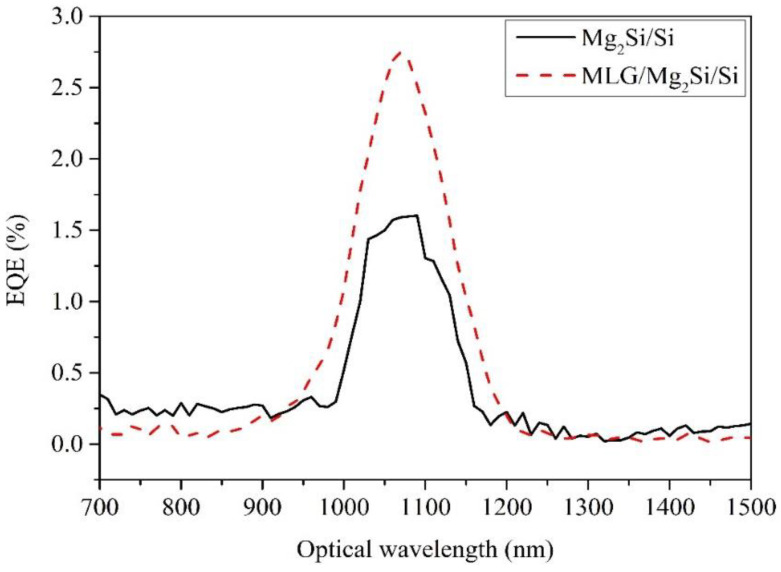
The EQE curves of Mg_2_Si/Si and MLG/Mg_2_Si/Si PDs.

The experimentally measured EQE curves for Mg_2_Si/Si and MLG/Mg_2_Si/Si heterojunction PDs are shown in Figure 7. The expression for the external EQE is given in Equation (2) [33,34], where *I_photo_*/*e* is the average number of photoelectrons generated per unit time, *P*/*hν* is the average number of incident light per unit time and *R* is the responsivity:

(2) $$EQE=\frac{I_{photo}/e}{P/h\nu }=\frac{h\nu }{e}R=\frac{hc}{e\lambda }R\;$$

The results exhibit that the peak EQE of the Mg_2_Si/Si heterojunction PD is 1.6%, and the MLG/Mg_2_Si/Si heterojunction PD is 2.75%. The peak wavelength is around 1090 nm, and the response wavelength is 700–1500 nm. Compared with the peak EQE of Mg_2_Si/Si PD, the peak EQE of MLG/Mg_2_Si/Si PD is enhanced and the enhancement ratio is about 71.87%. It can be noted from Equation (2) that the EQE is proportional to the responsivity under the condition of the same incident light wavelength. Compared with Mg_2_Si/Si PD, the responsivity of MLG/Mg_2_Si/Si PD is improved, so the EQE of MLG/Mg_2_SiSi PD is also improved.

After adding the MLG layer, a strong electric field is formed at the MLG/Mg_2_Si interface. This strong electric field is conducive to improving the effective separation and transfer of photogenerated carriers, which improves EQE [20]. At the same time, MLG, as a carrier transport layer, and its higher mobility are also conducive to improving the response rate and EQE [20]. Shevlyqgin et al. [14] measured the peak EQE of the Mg_2_Si/Si heterojunction near 850 nm, which was about 2.3%. After adding MLG, the peak EQE of PD is 2.75%, which exceeds the previous value of 2.3%, indicating that the technology of adding MLG to regulate the PD’s EQE is feasible.

### 3.6. Dark Current

The dark current of the Mg_2_Si/Si and MLG/Mg_2_Si/Si heterojunction PDs are measured using the Keithley 2614B digital source meter. The stable dark current of Mg_2_Si/Si PD is 1.26 nA, and that of MLG/Mg_2_Si/Si PD is 1.16 nA. The results show that the dark current of MLG/Mg_2_Si/Si PD decreases. After adding the MLG layers, MLG/Mg_2_Si/Si PD forms two barrier regions (MLG/Mg_2_Si and Mg_2_Si/Si), which are not conducive to carrier transport under the condition of no light. The weak p–type doping of MLG in the air causes the energy level of MLG to shift towards the valence band direction, thereby increasing the barrier height, and the transport of charge carriers becomes difficult in the absence of light. Overall, the dark current of the PD decreases after adding the MLG layer.

### 3.7. Other Detection Properties

The NEP expressed in Equation (3) is the incident light power when the signal output generated by the PD equals the square root of the noise output [35,36]. Where *P_incident_* is the incident light power, *I_photo_* is the output photocurrent, *I_N_* is the noise current, *I_dark_* is the dark current, *e* is the elementary charge and *R* is the responsivity.

(3) NEP=PincidentIphotoIN=INR=2eIdarkR

D* is defined as the reciprocal of *NEP*. Considering the different effective detection areas of PDs, D* could be expressed as shown in Equation (4) [37,38], where *S*, *e*, *I_dark_* and *R* are the effective working area, elementary charge, dark current and responsivity of heterojunction PD, respectively:

(4) $$D^{*}=\frac{\sqrt{S}}{\sqrt{2eI_{dark}}}R=\frac{\sqrt{S}}{NEP}$$

The on/off ratio refers to the ratio of the output signal of the photodetector under illumination conditions to the output signal under no illumination conditions, as shown in Equation (5) [39,40]. Where *I_light_* is the output current when illuminated, and *I_dark_* is the output current when there is no illumination:

(5) $$On/off\;\;ratio=\frac{I_{light}}{I_{dark}}$$

Based on the already measured responsivity, EQE and the dark current of the Mg_2_Si/Si and MLG/Mg_2_Si/Si heterojunction PDs, the NEP value can be obtained using Equation (3). The D* value can be obtained from Equation (4), and the on/off ratio can be obtained using Equation (5). The main detection properties of the Mg_2_Si/Si and MLG/Mg_2_Si/Si heterojunction PDs and other PDs are shown in Table 1. 

It can be seen that compared with the Mg_2_Si/Si heterojunction PD, the detection properties of the MLG/Mg_2_Si/Si heterojunction PD exhibit an overall enhancement. Its peak responsivity, peak EQE, peak NEP, peak D*, dark current and on/off ratios are 23.7 mA/W, 2.75%, 8.13 × 10^−13^ WHz^−1/2^, 0.12 × 10^11^ Jones, 1.16 nA and 4086, respectively, and their corresponding enhancement ratios are 60.56%, 71.88%, 40.22%, 62.16%, 8% and 74.54%, respectively. In this study, the peak responsivity (23.7 mA/W) and EQE (2.75%) of the MLG/Mg_2_Si/Si heterojunction PD are superior to the peak responsivity (10 mA/W) and EQE (2.3%), studied by Shevlyqgin [14]. It provides a direction for regulating the detection properties of Mg_2_Si/Si-based optoelectronic devices.

After adding MLG layers, the light absorption of the MLG/Mg_2_Si/Si heterojunction PD is enhanced, thereby improving the photocurrent, responsivity and EQE of the PD. Two barrier regions are formed at the MLG/Mg_2_Si and Mg_2_Si/Si interfaces, effectively reducing the dark current in the absence of illumination. Reducing the dark current benefits improving NEP and the on/off ratio. A high built-in electric field is formed at the MLG/Mg_2_Si interface, improving the ability of photogenerated carriers to separate and transfer when illuminated effectively. Compared with the Mg_2_Si/Si heterojunction PD, the MLG//Mg_2_Si/Si heterojunction PD illustrates an overall enhancement in the detection properties.

## 4. Conclusions

In this investigation, the Mg_2_Si/Si heterojunction is obtained by magnetron sputtering and annealing in the absence of argon or nitrogen atmosphere. MLG is transferred to the Mg_2_Si/Si heterojunction substrate by the suspended self-help transfer MLG method, and then the MLG/Mg_2_Si/Si heterojunction PDs is fabricated. The Mg_2_Si/Si and MLG/Mg_2_Si/Si heterojunctions are characterized by XRD, SEM and Raman spectra, and their responsivity, EQE and dark current are measured and analyzed. The experimental results show the successful fabrication of the Mg_2_Si/Si and MLG/Mg_2_Si/Si heterojunction PDs, along with achieving some detection capabilities. Among them, the optical absorption and effective separation and transfer of photogenerated carriers of the MLG/Mg_2_Si/Si heterojunction PD are improved, and the detection properties such as responsivity, EQE, NEP, D* and on/off ratio have been significantly enhanced.

The spectral range of the MLG/Mg_2_Si/Si heterojunction PD is 700–1500 nm, and its peak responsivity, peak EQE, peak NEP, peak D*, on/off ratio, dark current and photocurrent are 23.7 mA/W, 2.75%, 8.13 × 10^−13^ WHz^−1/2^, 0.12 × 10^11^ Jones, 4086, 1.16 nA and 4.74 μA, respectively. Compared with the Mg_2_Si/Si heterojunction PD, the enhancement ratios of peak responsivity, peak EQE, peak NEP, peak D*, on/off ratio, dark current and photocurrent of the MLG/Mg_2_Si/Si PD are 60.56%, 71.88%, 40.22%, 62.16%, 74.54%, 8% and 60.67%, respectively. Its peak responsivity and EQE are better than the earlier studies. The outcome of this study illustrates that the technology of introducing MLG to regulate the detection properties of the Mg_2_Si/Si heterojunction PD is feasible and provides a direction for regulating the detection properties of optoelectronic devices.

## Figures and Tables

**Table 1:** The main detection properties of Mg_2_Si/Si and MLG/Mg_2_Si/Si heterojunction PDs and other PDs.

	Responsivity (mW/A)	EQE (%)	NEP (WHz^−^^1/2^)	D* (Jones)	Dark Current (nA)	On/Off Ratio	Peak Wavelength (nm)	References
Mg_2_Si/Si	14.76	1.6	1.36 × 10^−12^	0.74 × 10^10^	1.26	2341	1090	this work
MLG/Mg_2_Si/Si	23.7	2.75	8.13 × 10^−13^	0.12 × 10^11^	1.16	4086	1090	this work
Graphene/Si	520		5.96 × 10^−15^	5.8 × 10^13^	1–10	2 × 10^7^	800–950	[41]
Graphene/Ge	51.8			1.4 × 10^10^		2 × 10^4^	1400	[42]
Graphene/GaAs	1.73			1.8 × 10^11^		10^4^	850	[43]

## Data Availability

Data are available on request.
